# Supplementary material for: The Discovery of Novel α2a Adrenergic Receptor Agonists Only Coupling to Gαi/O Proteins by Virtual Screening
Source: Int J Mol Sci. 2024 Jun 30;25(13):7233. doi: 10.3390/ijms25137233 (PMC11241340; doi:10.3390/ijms25137233)
Supplement: Supplementary file 1 [file ijms-25-07233-s001.zip › ijms-3008034-supplementary.pdf]

SUPPLEMENTARY MATERIALS

The discovery of novel α_{2A} adrenergic receptor agonists only coupling to Gαi/o proteins by virtual screening

Peilan Zhou*, Fengfeng Lu, Huili Zhu, Beibei Shi, XiaoxuanWang, Shiyang Sun, Yulei Li, RuibinSu*

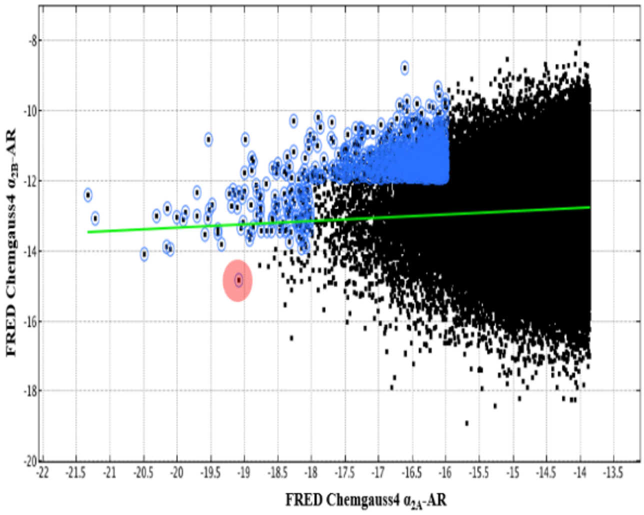

Figure S1. The plot of the docking scores of compounds to α_{2A}-AR and α_{2B}-AR. The compounds with high binding affinity to α_{2A}-AR but low binding affinity to α_{2B}-AR were colored by blue. C593-0297(red) showed high binding affinity to both α_{2A} and α_{2B}-AR.

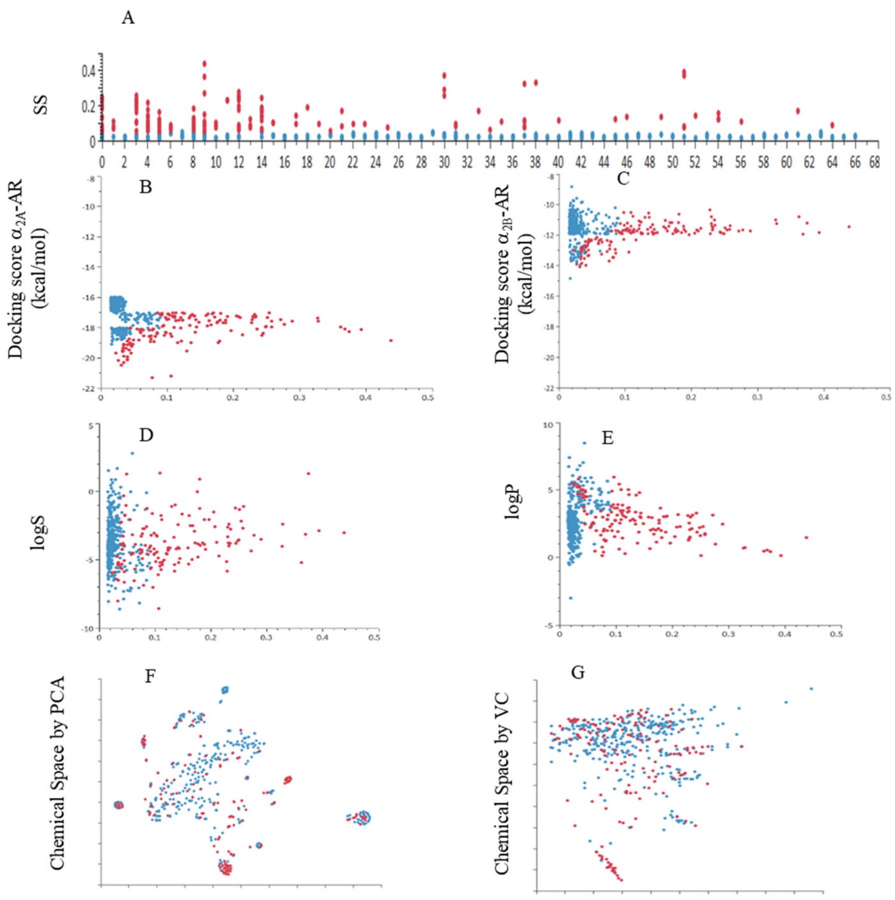

Figure S2 Correlation analysis of SS and other properties among 500 compounds

(A) Summarized scores (SS) of 500 compounds; (B) The docking scores of compounds to α_{2A}-AR compared to SS; (C) The docking scores of compounds to α_{2B}-AR compared to SS;(D) The LogS of compounds compared to SS; (E) The LogP of compounds compared to SS;(F) Chemical space by principal component analysis;(G) Chemical space by visual clustering. 140 compounds (red) were selected for further study.

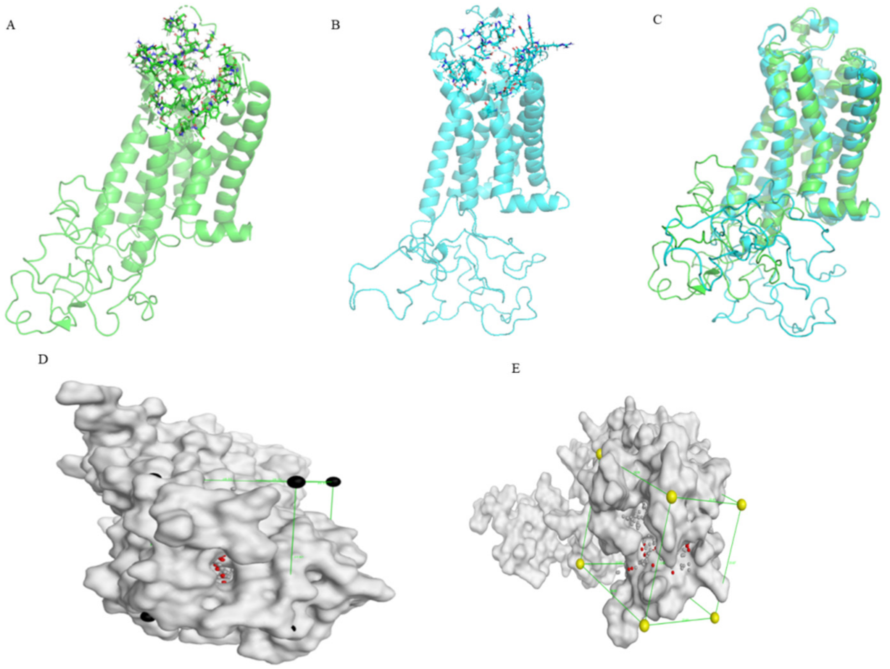

Figure S3 The 3D structure and docking box of α_2-AR

The 3D structure of α_{2A}-AR (A), α_{2B}-AR (B), the alignment of α_{2A} and α_{2B}-AR (C), the size and the residues of the docking box at the surface of α_{2A}-AR (D), the size and the residues of the docking box at the surface of α_{2B}-AR (E). The red and gray ball indicates the ligand binding area.

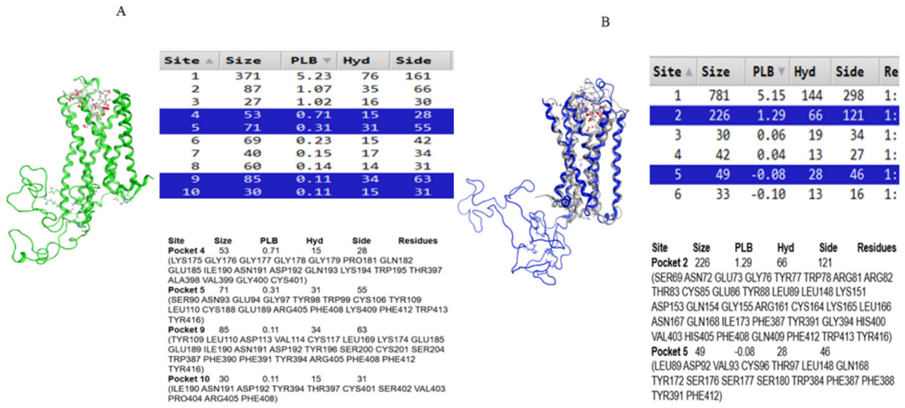

Figure S4 3D structure of α_{2A}-AR with the small molecular binding pocket (A) and α_{2B}-AR with the small molecular binding pocket (B).

Table S1 The docking scores and ADMET of the active compounds

IDNUMBER	FRED Chemgauss4 2A	FRED Chemgauss4 2B	logP	logSw	logS	HIA category	2D6 affinity category	PPB90 category	MW
E734-0162	-17.35	-11.51	0.15	-1.3	2.798	+	low	low	388.4
L470-0509	-17.91	-11.92	1.64	-3.51	1.91	+	medium	high	419.5
3254-4398	-18.52	-11.79	3.3	-1.19	1.395	+	low	high	389.4
G434-0357	-18.19	-11.98	1.09	-2.04	2.17	-	low	low	416.4
F784-0343	-18.88	-11.73	3.67	0.91	1.815	+	medium	high	398.5
S022-1543	-17.29	-11.91	1.164	-3.74	1.696	+	low	high	420.5
6428-0446	-17.65	-11.82	2.51	-1.63	0.5462	+	medium	high	343.2
D412-0085	-18.29	-11.93	3.04	-5.19	0.6125	+	medium	high	408.9
P278-0821	-17.84	-11.89	2.489	-5.83	1.646	+	high	high	349.4
Y031-0990	-17.35	-11.75	3.88	-5.72	1.422	+	medium	high	394.9
S020-2406	-17.98	-11.8	3.311	-1.753	0.4069	+	medium	high	392.4
C301-7899	-17.09	-11.83	3.77	-4.37	1.335	+	medium	high	379.4
G650-0551	-17.13	-11.74	2.29	-4.12	0.02211	+	medium	high	371.4
P300-2342	-17.47	-11.91	4.12	-5.44	1.547	+	medium	high	435.5
D272-0913	-18.42	-13.19	2.8	-4.55	1.883	+	medium	low	305.3
Y032-2014	-18.1	-11.73	2.11	-6.84	0.5807	+	high	high	458.5
Y041-2821	-21.32	-12.44	0.42	-4.88	1.7	-	low	low	378.4
D272-0919	-18.58	-13.09	3.46	-4.51	1.528	+	medium	high	339.8
Y030-6026	-17.08	-11.58	5.12	-7.09	0.737	+	medium	high	451.5
Y030-3813	-17.45	-10.72	2.29	-7.03	0.3968	+	high	high	472.5
F784-0278	-20.14	-12.82	4.71	-5.28	1.628	+	medium	high	384.5
F784-0364	-19.58	-13.56	4.75	-4.74	1.114	+	medium	high	359.4
Z56769139	-15.1363	-11.03	6.171	-6.43	6.171	+	medium	high	391.4
Z30249611	-11.9676	-8.56	0.613	-3.759	0.613	-	medium	low	275.3
